# Molecular Characterization and Phylogenetic Congruence of *Hydropsyche sciligra* (Tricoptera: Hydropsychidae) Using Mitochondrial and Nuclear Markers

**Published:** 2017-03-14

**Authors:** Naseh Maleki-Ravasan, Abbas Bahrami, Hassan Vatandoost, Mansoureh Shayeghi, Mona Koosha, Mohammad Ali Oshaghi

**Affiliations:** 1 Malaria and Vector Research Group (MVRG), Biotechnology Research Center (BRC), Pasteur Institute of Iran (PII), Tehran, Iran; 2 Department of Medical Parasitology and Mycology, Faculty of Medicine, Alborz University of Medical Sciences, Alborz Province, Karaj, Iran; 3 Department of Medical Entomology and Vector Control, School of Public Health, Tehran University of Medical Sciences, Tehran, Iran

**Keywords:** Caddisflies, *Hydropsyche sciligra*, COI, LSU rDNA, Molecular systematics

## Abstract

**Background:**

Caddisflies have significant roles in freshwater ecosystems. Morphological identification is the major impediment in accurate species identification of Hydropsychids. Mitochondrial and nuclear markers are suitable for molecular systematics of these group of arthropods.

**Methods:**

Trichopteran specimens of Lavasan District in northeastern Tehran, Iran were collected in 2012, and described using the morphological and molecular characters of mitochondrial cytochrome c oxidase subunit I (mt-COI) and three expansion fragments of large subunit (LSU) nuclear ribosomal DNA (28S rDNA) D1, D2, and D3. The resemblance of the specimen sequences was obtained by conducting BLAST searches against the GenBank database and by using simple maximum likelihood clustering using COI, D1, D2, D3, and combination of D1-D2-D3 sequence data sets.

**Results:**

Based on morphological traits the specimens were resembled to *Hydropsyche sciligra* however there were no its counterpart sequences in the GenBank. Due to lack of unique group of data set for each gene fragment, the specimens were associated with different taxa on molecular phylograms. The sequence contents of the COI, D1, D2, D3, and D1-D3 regions clustered *H. sciligra* with *H. brevis*, *H. angustipennis*, *H. occidentalis*, *H. hedini*, *H. grahami*, and *H. longifurca/H. naumanni,* respectively.

**Conclusion:**

Phylogenies obtained from combination of D1-D3 showed the highest bootstrap values for most of clades suggesting that long LSU-rDNA potentially is more useful for understanding phylogenetic relationships of caddisflies. A large-scale molecular and zoogeographic study on trichopteran species is suggested to revise and to develop the current knowledge of the caddisfly fauna and distributions in the country.

## Introduction

Hydropsychid caddisflies (Trichoptera: Hydropsychidae) have significant importance due to their role as biomonitoring indicators, immense geographical distribution, and their ecological position in aquatic food webs (Geraci et al. 2010, Maleki-Ravasan et al. 2013a). They also are important for human and animal health since they are sources of severe allergy. For example, their extensive exuviae reason inhalant allergens or the tiny setae of their wings and bodies may cause swelling and soreness in the eyes of people who encounter these potential allergens. In addition, the newly emerged adults of caddisflies may cause severe nuisance (Seshadri 1955, Fremling 1959, Corbet 1966).

To date, more than 1600 hydropsychid species have been described worldwide (Morse 2015). Genus *Hydropsyche* includes the most species lineages in all of Trichoptera order with more than 500 described species and are distributed in Holarctic, Oriental, Afrotropical, and Australasian streams and rivers (Morse 2015). *Hydropsyche* larvae exhibit a wide range of pollution tolerances (Resh and Unzicker 1975, Lenat 1993, Lenat and Resh 2001).

Freshwater biomonitoring which involves identifying the species inhabiting an ecosystem to provide an ongoing assessment of water quality, promises to be an efficient and cost-effective method to manage water resources particularly in the countries with low precipitations (Morse et al. 2007). Hence, species identification has become a prerequisite for any ecological study and biomonitoring approach. Moreover, larva identification is important for phylogenetic studies at higher level of trichopteran (Frania and Wiggins 1997).

Although Hydropsychid caddisflies are among the most frequently encountered macro-invertebrates in freshwater habitats and displays a wide range of tolerance values (Lenat 1993), however, their application in biomonitoring has been greatly impeded by the lack of identified and illustrated species, especially in countries such as Iran, where Trichoptera fauna was studied by non-autochthonous researchers (Schmid 1959, Malicky 1986, Mirmoayedi and Malicky 2002, Mey 2004, Malicky 2004, Chvojka 2006). Until recently, 62 trichopteran species were known from Iran (Morse 2015).

Morphological taxonomy of caddisflies is based on characters of adult male’s genitalia in association with its larva for species description and illustration at species level. Conventional approaches to larval association usually involve rearing larvae or morphological identification of metamorphotypes comprising mature pharate adult, larval sclerites, and pupal exuviae in the same pupal case (Milne 1938, Wiggins 1996).

Both approaches work well when adequate resources and expertise are available (Resh 1972, Floyd 1995, Glover 1996). However, these approaches have some limitations including larvae that develop into adults no longer exist as larvae, and descriptions must be made from similar (deemed identical) individuals. In addition, larval rearing is complicated by our imperfect understanding of species-specific microhabitat and water-chemistry requirements, particularly for some groups such as hydropsychids. Metamorphotypes are relatively rare because that portion of the life cycle occurs for a short time only, which means that chance encounters play a significant role in metamorphotype associations.

The molecular method for larval association could significantly accelerate the process of larval descriptions for a poorly known caddisfly fauna (Zhou et al. 2007). Recently, molecular methods have been developed for species determination and applied for different groups of insects at high or low level of phylogeny such as sand flies (Moin-Vaziri et al. 2007, Absavaran et al. 2009), mosquitoes (Oshaghi et al. 2003, 2006a, 2008, 2011, Mehravaran et al. 2011) and flies (Maleki-Ravasan et al. 2012). The main advantages of these methods are their sensitivity and specificity, independently of the stage, tissue or organ, live or dead of the specimen. The PCR-based species identification provides a convenient alternative for laboratories using primarily DNA-based techniques, and may be necessary when the study design already requires the use of individual DNA extractions for multiple purposes such as species confirmation, determination of food in predators (Morales et al. 2003, Sheppard et al. 2005, Oshaghi et al. 2006b, Maleki-Ravasan et al. 2009, Li et al. 2011, Sint et al. 2011), finding symbiont flora (Dale and Moran 2006, Russell et al. 2012, Chavshin et al. 2012, 2014, 2015, Maleki-Ravasan et al. 2013b, 2015), infection status for various pathogens (Oshaghi et al. 2009a, 2009b, 2010), and population genetic studies (Oshaghi et al. 2007).

Ribosomal DNA (rDNA) and cytochrome oxidase subunit I (COI) are the most widely used regions of the nuclear and mitochondrial genome, respectively to infer genetic variations and phylogenetic relationships for a vast group of organisms. Among the mitochondrion genes, the COI gene has been extensively used for phylogenetic analysis by itself or in combination with nuclear genes, and has proven to be phylogenetically highly informative in many insect groups including trichopterans (Whiting et al. 1997, Hyliš et al. 2007, Sonnenberg et al. 2007, Zhou et al. 2009, Ishiwata et al. 2011, Johanson et al. 2012, Ruiter et al. 2013).

In the present study, we aimed to provide and compare the sequences of three parts of rDNA (LSU rDNA D1, D2, D3) and COI genes for our poor morphologically identified caddisfly specimens and to develop phylogenetic topologies to identify or to bound species level for our caddisfly specimens.

## Materials and Methods

### Specimen collection

This study was conducted in summer time of 2012 in Lavasan River, northeastern Tehran, Iran. Immature stages of trichopteran insects were collected using D-frame nets and replacing stones from riverbed where water run, riffle, or stream bank and trichopteran larvae stick their retreat under or beside the stones. The retreats that might dock juvenile insect preserved in 70% ethanol and transferred to the School of Public Health (SPH) laboratory, Tehran University of Medical Sciences, Iran. The morphological characters of the extracted immature Trichoptera plus retreats general feature were used to species identification using the morphological key (Pescador et al. 1995) under microscope (Olympus SZX12).

### DNA extraction, PCR, and sequencing

Genomic DNA from larva and pharate adult was extracted using Qiagen DNeasy Tissue Kit (Qiagen, Hilden, Germany), which uses silica to bind DNA. The mt-COI gene extending 690bp of 5’ fragment as applied by (Lunt et al. 1996) was amplified using primers of C1-J-2090 and C1-N-2735 (Table 1).

**Table 1.** Details of primers and PCR products used for amplification of caddisfly mitochondrial and nuclear genes

**Gene Name**		**Primer name**	**Sequence (5’ to 3’)**	**PCR product (bp)**	**Reference**
**mt-COI**	COI	C1-J-2090	AGTTTTAGCAGGAGCAATTACTAT	∼690	(Zhang and Hewitt 1997)
		C1-N-2735	AAAAATGTTGAGGGAAAAATG TTA		
**nrDNA**	D1	D1–UP	GGAGGAAAAGAAACTAACAAGGATT	∼330	(Geraci et al. 2010)
		D1–DN	CAACTTTCCCTTACGGTACT		
	D2	D2-UP	GAGTTCAAGAGTACGTGAAACCG	∼430	
		D2-DN	CCTTGGTCCGTGTTTCAAGAC		
	D3	D3–UP	ACCCGTCTTGAAACACGGAC	∼230	
		D3–DN	CTATCCTGAGGGAAACTTCGGA		

The amplification was performed in 20μl reactions in premix ready to use kits under two thermal circulations. The first circulation started after initial denaturation at 94 °C for 2min, as follows: 5 cycles of 94 °C for 40s, 45 °C for 40s, and 72 °C for 1min. The second thermal cycle was repeated for 35 cycles for 94 °C for 40 s, 51°C for 40 s, and 72°C for 1 min followed by a final extension step at 72 °C for 5 min. Amplification of the nrDNA fragments was performed using 1μL of genomic DNA from each specimen in 20-μl reactions. The PCR mix was preheated at 94 °C for 3min followed by 40 cycles of 94 °C for 30s 60 °C for 45s, and 72 °C for 60 s. After 10min of final extension at 72 °C, the products were maintained at 4 °C.

PCR products were visualized on a 1% agarose gel containing ethidium bromide using an UV transilluminator. The PCR products were directly sequenced by Seqlab (Guttenberg, Germany). Sequences from both directions were aligned and proofread with the program ChromasPro (version 1.2, Windows, Technelysium Pty Ltd, Tewantin, Queensland, Australia). Basic Local Alignment Search Tool (BLAST) (Altschul et al. 1997) was used to compare the nucleotide sequences with data of NCBI database and to make sure correct fragment amplification. Sequences of mt-COI and nrDNA regions were aligned with CLUSTALW as implemented in BioEdit (Hall 1999).

### Phylogenetic Analysis

For phylogenetic analysis the sequences obtained in this study was combined with all of the D1, D2, D3, and COI sequences of the Hydropsychid caddisflies available in GenBank (Table 2) (https://www.ncbi.nlm.nih.gov/genbank/). Due to the different lengths of the sequences, they were trimmed to obtain a consistent region for phylogenetic analysis. Pairwise sequence divergence, using Kimura’s two-parameter distance algorithm, and the maximum likelihood trees presented herein were processed in MEGA 5.0 (Tamura et al. 2011). To combine three rDNA (D1, D2, D3) fragments we have to refine our analysis to the species that their sequences were available for the three fragments (Table 3). Phylogenetic analyses were performed on various datasets, including DNA of D1, D2, D3, and COI separately and combination of D1–D3 fragments. The reliability of the branching order was determined by 1000 bootstrap replications (Felsenstein 1985).

**Table 2.** Details of GenBank sequence data used for phylogenetic analysis. The two first rows obtained in this study

**Nuclear Large Subunit rRNA [28S]**			**Mitochondrial Cytochrome Oxidase subunit I [mt-COI]**
**D1**	**D2**	**D3**	
*Hydropsyche sciligra* LD11 (JX419391)	*Hydropsyche sciligra* LD12 (JX419393)	*Hydropsyche sciligra* LD13 (JX419395)	*Hydropsyche sciligra* L190 (JX419389)
*Hydropsyche sciligra* PAD1 (JX419392)	*Hydropsyche sciligra* PAD2 (JX419394)	*Hydropsyche sciligra* PAD3 (JX419396)	*Hydropsyche sciligra* PA90 (JX419390)
*Hydropsyche angustipennis* (EF417120)	*Hydropsyche angustipennis* (EF417120)	*Parapsyche elsis* (AF436341)	*Hylesinus fraxini* (HM002626)
*Wormaldia triangulifera* (AF436226)	*Mexipsyche furcula* (EF513990)	*Wormaldia triangulifera* (AF436345)	*Hydropsyche fezana* haplotype 02 (HM134822)
*Hydropsyche occidentalis* (AF436212)	*Herbertorossia quadrata* (EF513918)	*Hydropsyche occidentalis* (AF436332)	*Hydropsyche fezana* haplotype 01 (HM134821)
*Cheumatopsyche lepida* (EF417118)	*Cheumatopsyche lepida* (EF417118)	*Arctopsyche grandis* (AF436342)	*Hydropsyche pellucidula* haplotype 10 (HM134819)
*Cheumatopsyche oxa* (AF436213)	*Caledopsyche* sp. (EU254421)	*Caledopsyche sp*. (EU254458)	*Hydropsyche pellucidula* haplotype 06 (HM134815)
*Plectropsyche hoogstraali* (HM167451)	*Potamyia flava* (HM167448)	*Hydropsyche cf. grahami* (EU254462)	*Hydropsyche pellucidula* haplotype 09 (HM134818)
*Calosopsyche continentalis* HM167450	*Hydromanicus sp.* (EF513893)	*Hydropsyche sparna* (HSU65201)	*Hydropsyche incognita* haplotype 03 (HM134807)
*Mexipsyche cf grahami* (EU312009)	*Mexipsyche nr rhomboana* (EF513991)	*Smicridea* sp. (EU254467)	*Hydropsyche incognita* haplotype 04 (HM134808)
*Orthopsyche fimbriata* (EU250332)	*Hydropsychehedini* (EF513985)	*Orthopsyche thomasi* (EU254468)	*Hydropsyche incognita* haplotype 02 (HM134806)
*Homoplectra flinti* (EU312025)	*Hydropsyche instabilis* (HM167440)	*Maesaipsyche* sp. (EU254474)	*Hydropsyche incognita* haplotype 01 (HM134805)
*Aoteapsyche colonica* (AF436215)	*Hydropsyche botosaneanui* (HM167436)	*Trichoptera environmental* (DQ086620)	*Hydropsyche incognita* haplotype 05 (HM134809)
*Hydropsyche naumanni* (EU312012)	*Hydropsyche siltalai* (HM167444)	*Diplectrona zealandensis* (EU254475)	*Hydropsyche brevis* (JQ687920)
*Hydropsyche longifurca* (EU312026)	*Mexipsyche nr rhomboana* (EF514025)	*Smicrophylax* sp. (EU254457)	*Hydropsyche fezana* (JQ687901)
*Ceratopsyche bronta* (AF436214)	*Herbertorossia* sp. (EF514022)	*Aoteapsyche colonica* (AF436335)	*Hydropsyche lobata* (KF255638)
*Cheumatopsyche afra* (EU312016)	*Mexipsyche nr rhomboana* (EF513992)	*Hydropsyche longifurca* (EU254472	*Hydropsyche exocellata* (KF255625)
*Diplectrona metaqui* (EU312024)	*Hydropsychesaxonica* (HM167443)	*Hydropsyche naumanni* (HM167457)	*Hydropsyche dinarica* (KF255619)
*Hydatopsyche melli* (EU312008)	*Mexipsyche nr rhomboana* (EF513994)	*Ceratopsyche bronta* (AF436334)	*Hydropsyche instabilis* (KF255636)
*Hydromanicusu mbonatus* (EU312010)	*Hydropsyche longifurca* (EU254450)	*Homoplectra flinti* (EU254471)	*Hydropsyche maroccana* (JQ687916)
*Streptopsyche parander* (HM167449)	*Aoteapsychecolonica* (HM167438)	*Diplectrona metaqui* (EU254470)	*Hydropsyche teruela* (KF255660)
*Orthopsyche Thomasi* (EU31202228)	*Hydropsyche naumanni* (EU254434)	*Streptopsyche parander* (HM167453)	*Hydropsyche modesta* (JQ687913)
*Cheumatopsyche triangularis* (EU312013)	*Ceratopsyche bronta* (HM167437)	*Hydatopsyche melli* (EU254461)	*Hydropsyche bulbifera* (JQ687900)
	*Diplectrona metaqui* (EU254448)	*Cheumatopsyche afra* (EU254465)	*Hydropsyche tibialis* (JQ687923)
	*Homoplectra flinti* (EU254449)	*Hydromanicus umbonatus* (EU254463)	
	*Streptopsyche parander* (EU254455)	*Hydropsyche exocellata* (JQ687958)	
	*Hydromanicusumbonatus* (EU254432)	*Hydropsyche instabilis* (JQ687954)	
	*Hydatopsyche melli* (EU254430)	*Hydropsyche pellucidula* (JQ687950)	
	*Cheumatopsyche afra* (EU254438)	*Hydropsyche modesta* (JQ687949)	
	*Mexipsyche nr grahami* (EF514002)	*Hydropsyche siltalai* (JQ687948)	
	*Hydropsyche nr formosana* (EF513958)	*Hydropsychefontinalis* JQ687947	
	*Ceratopsyche serpentine* (EF513917)	*Hydropsyche infernalis* (JQ687946)	
		*Hydropsyche fezana* (JQ687939)	
		*Hydropsyche lobata* (JQ687929)	
		*Hydropsyche tibialis* (JQ687962)	
		*Hydropsyche dinarica* (JQ687959)	
		*Hydropsyche incognita* (JQ687951)	
		*Hydropsyche brevis* (JQ687932)	
		*Hydropsyche teruela* (JQ687961)	
		*Hydropsycheiberomaroccana* (JQ687937)	
		*Cheumatopsyche lepida* (JQ687965)	
		*Hydropsyche bulbifera* (JQ687928)	

**Table 3.** Details of the GenBank sequence data used for phylogenetic analysis of rDNA D1-D2-D3 loci

**Species**	**Country**	**GenBank accession numbers**		
		**28S D1**	**28S D2**	**28S D3**
***Hydropsyche sciligra* (Larvae)**	Iran	JX419391	JX419393	JX419395
***Hydropsyche sciligra* (Pharate adult)**	Iran	JX419392	JX419394	JX419396
***Streptopsyche parander***	Dominican	HM167449	EU254455	HM167453
***Aoteapsyche colonica***	New Zealand	AF436215	HM167438	AF436335
***Hydropsyche naumanni***	Indonesia	EU312012	EU254434	HM167457
***Hydromanicus umbonatus***	China	EU312010	EU254432	EU254463
***Hydatopsyche melli***	China	EU312008	EU254430	EU254461
***Diplectrona metaqui***	USA	EU312024	EU254448	EU254470
***Ceratopsyche bronta***	USA	AF436214	HM167437	AF436334
***Homoplectra flinti***	USA	EU312025	EU254449	EU254471
***Hydropsyche longifurca***	Southeast Africa	EU312026	EU254450	EU254472
***Cheumatopsyche afra***	South Africa	EU312016	EU254438	EU254465
***Cheumatopsyche lepida***	West Palearctic	EF417118	EF417118	JQ687965

## Results

The specimens were resembled to *Hydropsyche sciligra* (Malicky 1977 Synonym: *H. gracilis* Martynov, 1909).

PCR amplification was successfully performed for the mitochondrial and nuclear genes for the specimens as outlined in the material and method section. The lengths of PCR products were roughly 690bp for COI, and 330, 430, and 230bp for D1, D2, and D3 of LSU, respectively. The generated sequences were deposited in GenBank database with accession numbers JX419389-96. The lengths of fragments used for phylogenetic analysis were 570bp for COI, 269bp for D1, 397bp for D2, 162bp for D3, and 828bp for D1-D3. Sequence information of the data obtained in this study and the data retrieved from GenBank database for each fragment or combined dataset are summarized in tables 2 and 3 respectively.

Cytochrome oxidase subunit I sequences were obtained for two specimens from Iran and 15 species from GenBank. COI length of the two specimens was 619bp, with three substitutions and their GC contents were 31% that is in agreement with known adenine/thymine (A/T)-rich content of mitochondrial genes. D1 sequences were obtained for two specimens from Iran and compared with 21 species from GenBank. The D1 sequence length of both LD11 and PAD1 samples were 307bp with 8 substitutions and 56 and 57% GC contents respectively. D2 sequences of the Iranian specimens compared with 27 species from GenBank. The D2 sequence length of both LD12 and PAD2 samples were 419 bp with three substitutions and their GC contents were 66%. D3 sequences were obtained for the specimens from Iran and compared with 40 species from GenBank. The D3 sequence lengths of both samples were 318 bp with six substitutions and their 55–56% GC contents. D1–D3 sequences were obtained for the specimens and compared with 11 species from GenBank. The D1–D3 sequence lengths of both specimens were 1044 bp with 17 substitutions and 60% GC content.

**Fig. 1.**
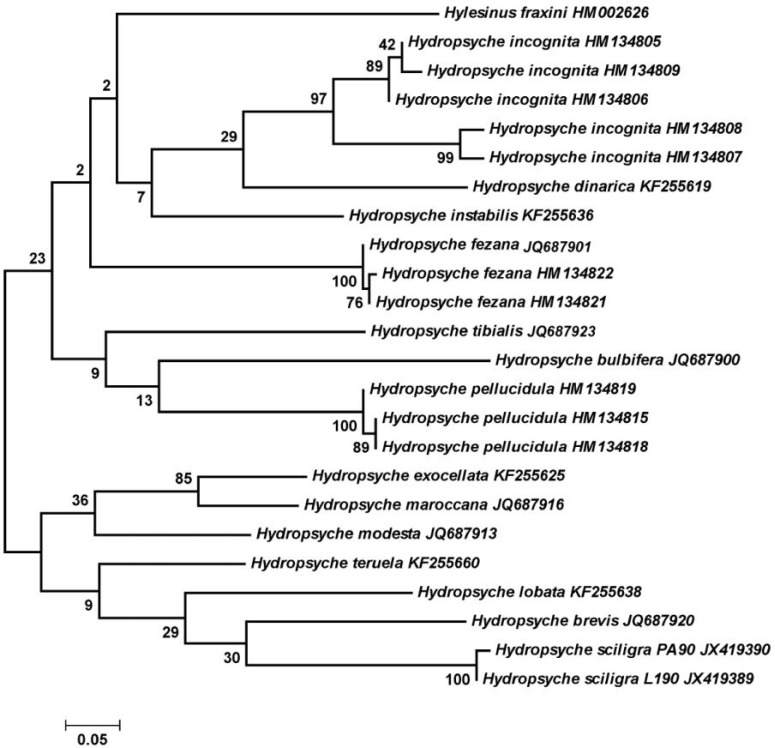
Phylogenetic relationship of Hydropsychid caddisflies inferred from 570bp of the mt-COI gene. Iranian samples are shown as JX419389-90. The bark beetle *Hylesinus fraxini* (Panzer, 1779) (Coleoptera: Scolytidae) used as out-group. Bootstrap values are shown at nodes. The scale of genetic distance is shown underneath

**Fig. 2.**
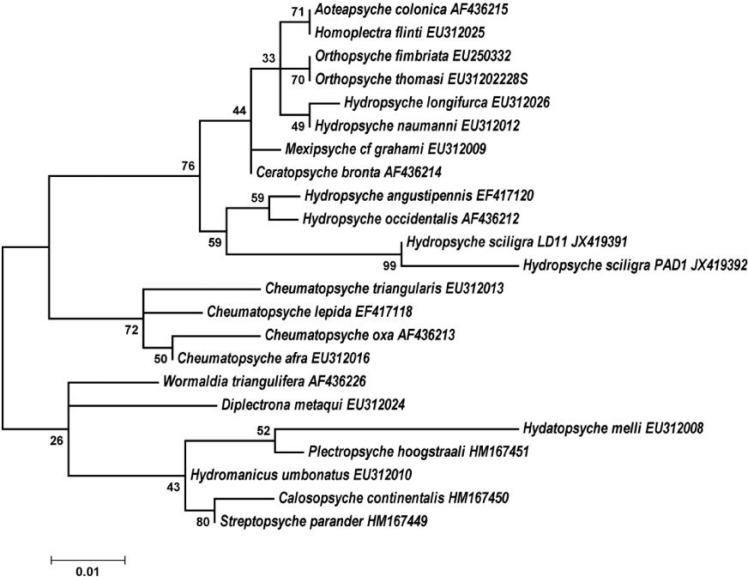
Phylogenetic relationship of Hydropsychid caddisflies inferred from 269bp of the 28S-D1-rDNA gene. Iranian samples are shown as JX419391-92. Bootstrap values are shown at nodes. The scale of genetic distance is shown underneath

Phylogenetic relevance based on COI sequence data showed affinity of the Iranian *H. sciligra* to *H. brevis* from West Palearctic ecozone with 30% bootstrap value (Fig. 1). The maximum likelihood tree topology based on D1 sequence data revealed that the Iranian *Hydropsyche* specimens were most closely related to *H. occidentalis* from Nearctic ecozone and *H. angustipennis* from East/West Palearctic ecozone with 59% support (Fig. 2).

**Fig. 3.**
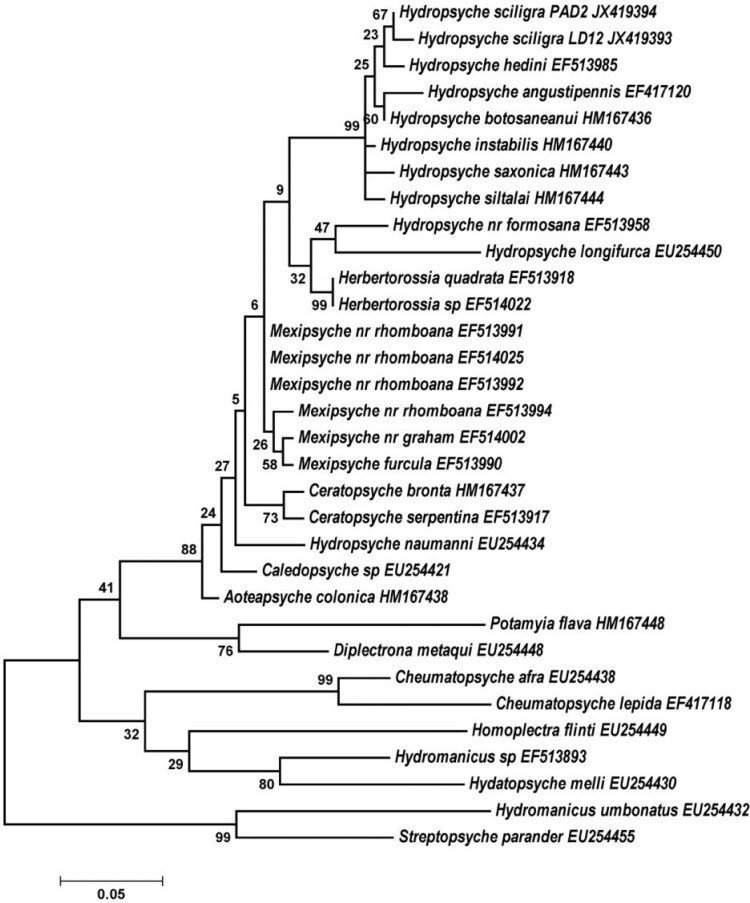
Phylogenetic relationship of Hydropsychid caddisflies inferred from 397bp of the 28S-D2-rDNA gene. Iranian samples are shown as JX419393-94. Bootstrap values are shown at nodes. The scale of genetic distance is shown underneath.

Sequence analysis of D2 fragment revealed that the Iranian *H. sciligra* were associated with *H. hedini* from Oriental ecozone with 23% bootstrap value. However, these pair species were associated with most of *Hydropsyche* including *H. angustipennis*, *H. botosaneanui*, *H. instabilis*, *H. siltalai* and *H. saxonica* from West Palearctic ecozone and formed a main clade with 99% support (Fig. 3).

**Fig. 4.**
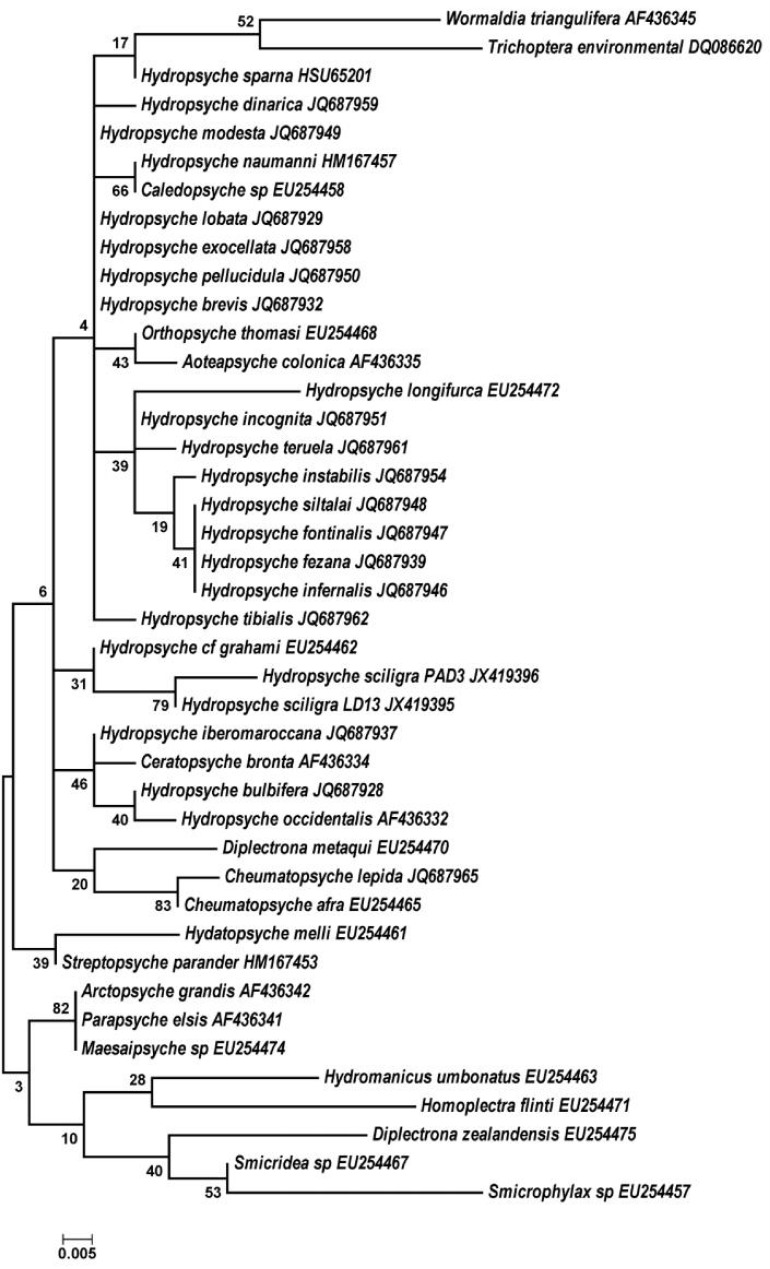
Phylogenetic relationship of Hydropsychid caddisflies inferred from 162bp of the 28S-D3-rDNA gene. Iranian samples are shown as JX419395-96. Bootstrap values are shown at nodes. The scale of genetic distance is shown underneath.

**Fig. 5.**
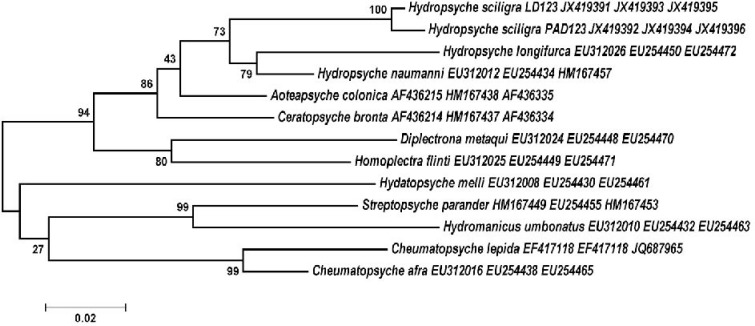
Phylogenetic relationship of Hydropsychid caddisflies inferred from 828bp of the 28S-D1-D2-D3-rDNA gene. Iranian samples are shown as JX419391-96. Bootstrap values are shown at nodes. The scale of genetic distance is shown underneath

Tree topology based on D3 sequence data showed an association between the Iranian *H. sciligra* and *H. cf graham* from Oriental part with only 31% support (Fig. 4). Phylogenetic analysis using the combined dataset of D1-D3 fragments recovered the Iranian *H. sciligra* in affinity with *H. longifurca* from Southeast Africa and *H. naumanni* from Indonesia with 73% support (Fig. 5). Generally, the bootstrap values were higher for long fragment of LSU than the individual fragments of LSU or even COI gene. However, the D2 fragment support strongly the monophyly of most *Hydropsyche* species including *H. sciligra*, *H. botosaneanui*, *H. angustipennis*, *H. hedini*, *H. instabilis*, *H. siltalai*, and *H. saxonica.* Phylogenetic congruence of Iranian *H. sciligra* based on different genes and their worldwide distribution are shown in Table 4.

**Table 4.** Details of phylogenetic congruence of Iranian *Hydropsyche sciligra*

**Gene**	**Putative species (Accession number)**	**Biogeographic Ecozone**
**COI**	*H. brevis* (JQ687920)	West Palearctic (France)
**D1**	*H. occidentalis* (AF436212)	Nearctic
	*H. angustipennis* (EF417120)	East Palearctic, West Palearctic (Netherlands, Belgium, Germany, Sweden, United Kingdom, Luxembourg, Norway, Finland, France, Austria, Czech Republic, Italy, Denmark, Russia, Slovenia, Hungary, Croatia, Isle of Man, Switzerland, Ireland, Greece, Macedonia)
**D2**	*H. botosaneanui* (HM167436)	West Palearctic (Greece, Belgium, Luxembourg, Germany France, Netherlands, Italy, Monaco)
	*H. angustipennis* (EF417120)	Like above
	*H. hedini* (EF513985)	Oriental (China)
	*H. instabilis* (HM167440)	West Palearctic (Europe and Northern Asia (excluding China))
	*H. siltalai* (HM167444)	West Palearctic: Europe and Northern Asia (excluding China) (Norway, Sweden, Finland)
	*H. saxonica* (HM167443)	West Palearctic: Europe and Northern Asia (excluding China) Germany
**D3**	*H. cf. grahami* (EU254462)	Oriental (China)
**D1-D2-D3**	*H. longifurca* (EU312026, EU254450, EU254472)	Afrotropical (South Africa, Lesotho, Zimbabwe, Swaziland)
	*H. naumanni* (EU312012, EU254434, HM167457)	Oriental (Indonesia)

## Discussion

In this study, we found only samples of one species *H. sciligra* in Lavasan district located in northeastern of Tehran. This species is widespread in Iran, Turkey and Caucasus (Morse 2015). This species has previously been reported from various parts of northern Iran including Chalus, Makou, Qazvin, Minoudasht, and northern parts of Alborz Mountains Chain (Mirmoayedi and Malicky 2002, Ivanov 2011). The discovery in Lavasan indicates that the dispersal area of this species is wider than currently known. Besides of this species, there are twelve species of *Hydropsyche* previously reported from certain provinces or regions of Iran and neighboring countries as follows: *H. consanguinea*, *H. demavenda*, *H. djabai*, *H. mahrkusha*, *H. ressli*, *H. sakarawaka*, *H. supersonica*, *H. iokaste*, *H. bujnurdica*, *H. esfahanica*, *H. lundaki*, and *H. masula* (Morse 2015).

Mitochondrial genes (mtDNA) particularly COI are used most frequently in different phylogenetic levels of trichopteran including order, families, subfamilies, genera, and species levels (Myers et al. 2001, Kjer et al. 2001, 2002, Johanson 2007, Malm and Johanson 2008, Pauls et al. 2008, Previšić et al. 2009, Johanson et al. 2009, Johanson and Malm 2010, Johanson and Espeland 2010, Espeland and Johanson 2010a, Espeland and Johanson 2010b, Malm and Johanson 2011). However, in this study bootstrap values of phylogenetic tree nodes were not enough high to support strongly the caddisflies relationship. It reflects lack of enough available data in GenBank than the phylogenetic utility of the gene.

In this study, 28S nrDNA was selected due to the high frequent available sequence data for trichoperan species in GenBank, which has provided good opportunity to compare our data with other trichopteran species. Nuclear ribosomal DNA belongs to a multigene family, where hundreds to thousands of copies of the nrDNA unit appear in tandem along the chromosome. Although individual fragments of the rDNA did not support well the topology of branches and clades in the trees, however, combination of three parts of the gene revealed the highest bootstrap values for the constructed trees. The combination of the three fragments (D1-D3) revealed 73% support value for association of *H. sciligra* with *H. longifurca* and *H. naumanni*. However, the limited number of trichopteran species (n=11) involved in the study may decline power of this analysis.

Between the COI and individual LSU fragments, D2 fragment strongly supported the monophyly of most *Hydropsyche* species. The D2 expansion fragment of 28S ribosomal RNA (rRNA) is one of the most highly variable regions in eukaryote rRNA. The length and nucleotide composition of this fragment is highly variable among insects (Gillespie et al. 2004). These significant variations limited the utility of D2 in deep-level phylogeny because of difficulties in alignment, although universally conserved RNA secondary structures have provided solutions for some taxa (Gillespie et al. 2004).

## Conclusion

Many areas in Iran have not been or poorly investigated for caddisfly fauna. Hence, a large-scale zoogeographic study using morphological and molecular characters comprising mitochondrial and nuclear markers together with population level sampling of all nominal taxa of trichopteran in poorly investigated areas of the country is highly suggested. These studies will revise and improve the current knowledge of the caddisfly distributions of the country and will enable better-applied strategies in protection for this beneficial group of aquatic insects.
